# Cell line bias in virus research: implications for viral propagation and biological interpretation

**DOI:** 10.3389/fcimb.2025.1675100

**Published:** 2026-01-09

**Authors:** Ji-Young V. Kim, Bradley S. Pickering

**Affiliations:** 1 National Centre for Foreign Animal Diseases, Canadian Food Inspection Agency, Winnipeg, MB, Canada; 2 Department of Medical Microbiology and Infectious Diseases, University of Manitoba, Winnipeg, MB, Canada

**Keywords:** cell line use in virology, virus propagation, virus characterization, cell line expression, cell line bias

## Abstract

Cell lines are essential tools in virology for propagating viruses for characterization studies. However, reliance on a few historically popular lines—such as Vero, BHK-21, and MDCK—can introduce bias and obscure important aspects of viral biology, such as entry mechanisms and replication dynamics. A review of over 6,000 publications revealed that a small number of cell lines are used disproportionately, often due to historical precedence and general permissiveness for viral infection. Gene expression analysis showed that while these lines are enriched for pro-viral process genes, many underutilized cell lines from diverse tissue types also exhibit similar profiles. This review calls for a more strategic, molecularly informed approach to cell line selection, including the development of molecular databases for non-human cell lines, identification of virologically relevant traits, and broader use of biologically diverse panels. Such a data-driven strategy is especially vital for studying emerging and zoonotic viruses, where accurate modeling of host–virus interactions is important. Expanding and refining cell line use will improve reproducibility and yield more accurate insights into viral pathogenesis.

## Introduction

In virus research, cell lines serve as the primary platform for viral propagation, enabling the study and characterization of viruses through various downstream applications. Despite their important role, little attention is paid to the impact of the choice of cell line. There are known examples that highlight how the choice of cell line can bias experimental outcomes and hinder accurate understanding of viral pathogens.

For instance, early Human immunodeficiency virus (HIV) research relied heavily on established T-cell lines such as H9 and MT-4 (Pauwels et al., 1987; Popovic et al., 1984). These cell lines selectively supported the growth of T-cell-tropic HIV variants, while failing to propagate macrophage-tropic strains that are predominant during early infection. This cellular bias delayed the recognition of C-C chemokine receptor 5 (CCR5) as a key co-receptor and inadvertently shaped research focus toward T-cell-tropic variants (Littman, 1998). Only after diversifying cellular models did the field begin to capture the full spectrum of HIV transmission, tissue tropism, latency and pathogenesis.

SARS-CoV-2 research presents a more recent example of how cell line selection affects viral characteristics. During the early pandemic phase, Vero E6 cells were widely adopted for viral isolation due to their availability and general permissiveness. However, these cells lack Transmembrane serine protease 2 (TMPRSS2), which is present in human airway epithelial cells and affects viral entry pathways (Matsuyama et al., 2020). Additionally, propagation in Vero E6 cells induced mutations in the spike protein that altered properties relevant to immune escape and pathogenesis (Liu et al., 2021). Recognition of these limitations prompted researchers to adopt more physiologically relevant systems, including human airway and intestinal epithelial cell lines, which better preserved viral characteristics.

In our own work with Rift Valley fever virus, we observed that a 78 kDa glycoprotein can only be observed in virus propagated in the mosquito-derived C6/36 cells and not in those grown in mammalian Vero E6 cells (Weingartl et al., 2014). This glycoprotein is suspected to play a role in vector-host transmission. This observation highlights that cellular environments can influence viral protein expression and potentially affect virus behaviors that could be important for zoonotic transmission.

These examples demonstrate that cell lines can introduce systematic biases and obscure important biological aspects of viral behavior. While testing multiple virus-cell line combinations could address these limitations, such approach is often impractical and costly. An optimal strategy should balance the efficiency of standardized systems with sufficient biological diversity to capture an array of virus behavior.

In this review, we examine commonly used cell lines for virus propagation and analyze their gene expressions, and compare them to other well established cell lines for permissiveness to viral infection. We argue that strategic selection of cell lines—based on both their ability to support virus growth and biological diversity—can improve the accuracy and relevance of virology studies without significantly increasing operational burden.

## Results

### Popular cell lines of choice for growing viruses

**Figure 1:**
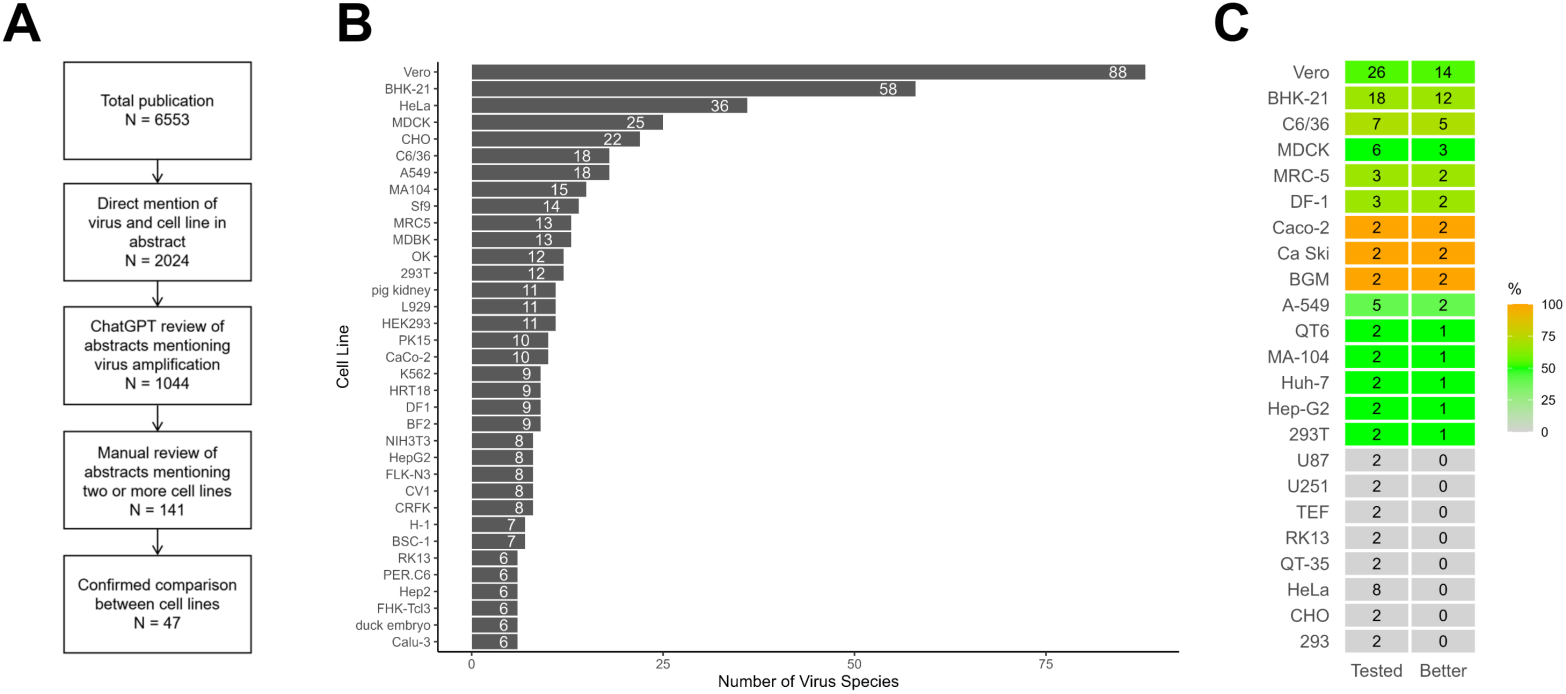
Schematic of the literature review and results. **(A)** Schematic of how the literature review was conducted. **(B)** Summary of commonly used cell lines based on literature review. The number of unique virus species reported to be propagated in each cell line is shown on the left. Values within the bars indicate the count of distinct virus species. **(C)** Summary of cell line performance in comparative virus growth studies. The “Tested” column indicates the number of publications in which each cell line was evaluated. The “Better” column reflects the number of studies where the cell line demonstrated superior viral growth performance compared to others. Cell lines are color-coded based on the percentage of favorable outcomes (Better divided by Tested), with green and blue indicating higher performance and orange indicating lower performance, as shown in the accompanying color scale.

The American Type Culture Collection (ATCC) catalogs over 4000 cell lines commercially available for research. However, their utilization and preference is likely not equally distributed. This was true for cell lines being used to grow viruses. Through a literature review, we identified 625 unique cell lines used to propagate 518 virus species. Among these, Vero cell line was most frequently used, while influenza virus was the most extensively studied (**Figure 1B**). For this analysis, all sublineages of Vero cell lines such as E6 and 76 were treated as one grou A more detailed analysis revealed virus-specific preferences for certain cell lines. For instance, MDCK cells showed strong association with influenza research, while Vero cells demonstrated broader utilization across diverse virus species, indicating their general permissiveness for viral propagation (data not shown).

We further examined studies that directly compared virus amplification efficiency across two or more cell lines. While such comparative analyses were uncommon and often involved limited cell line selections, several patterns emerged from available data. Cell lines such as Vero, BHK-21, and C6/36 consistently produced higher viral yields in more than half of the comparative studies in which they were evaluated (**Figure 1C**). Interestingly, HeLa cells consistently showed poorer performance across all eight publications that included this cell line in comparative analyses.

### Attributes of the popularly used cell lines

To assess whether those popularly used cell lines truly provide superior support for viral growth, we analyzed expressions of genes associated with viral processes in Vero E6, BHK-21 and MDCK. Using publicly available RNA expression datasets, we compared Vero E6, BHK-21, and MDCK cells to other well established human kidney cell lines. Kidney datasets were used as comparators because all three of the popularly used cell lines were also originated from kidneys in non-human organisms. C6/36 cells were excluded from this analysis due to difficulties in mapping *Aedes albopictus* gene expression data to human orthologs.

**Figure 2:**
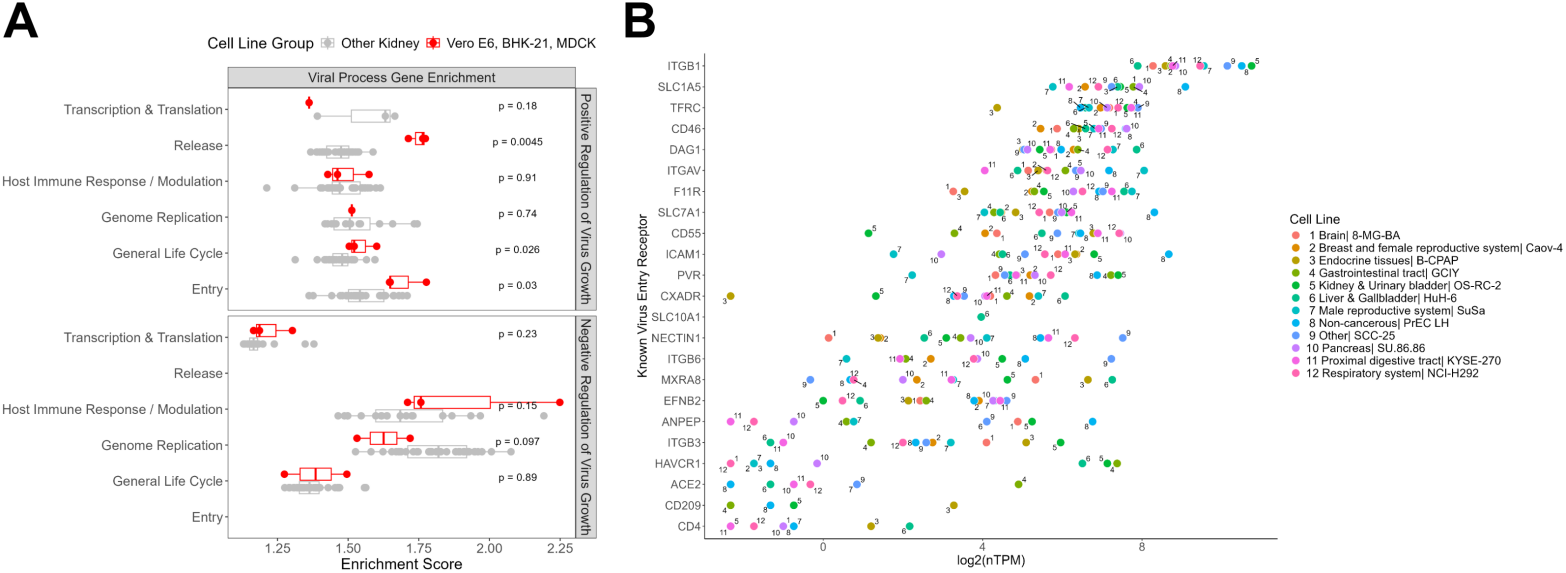
Gene expression profiles of commonly used cell lines in virus research. **(A)** Enrichment analysis of gene sets related to the positive and negative regulation of virus growth in popularly used cell lines (Vero E6, BHK-21, MDCK; shown in red) compared to other kidney-derived cell lines from the Human Protein Atlas (gray). The y-axis shows subcategories within each regulation group; the x-axis displays enrichment scores, with higher values indicating stronger gene set enrichment.**(B)** Known virus receptor expression in select cell lines. One cell line from each tissue type was selected for representation based on its overall expression similarity and viral receptor coverage to the popular cell lines. The y-axis shows known virus entry receptors, and the x-axis shows their normalized expression levels in each cell line.

We found that popular cell lines were enriched for genes involved in the positive regulation of viral processes, such as viral entry and release (**Figure 2A**). In contrast, we did not observe any statistical difference in enrichment for genes associated with negative regulation of viral growth between the popular and comparator cell lines. This observation may reflect the fact that the expression dataset used here was derived from baseline, unstimulated conditions, where antiviral responses were not engaged and therefore not detectable.

## Discussion

Despite the widespread use of established cell lines in virus research, our understanding of how these systems influence virus characteristics remains limited. Addressing this knowledge gap presents several opportunities to strengthen the rigor, efficiency, and biological relevance of future studies.

### Expanding molecular datasets for non-human cell lines

A significant limitation in current approaches is the lack of comprehensive molecular datasets for non-human cell lines, such as those derived from pigs, birds, or insects, which are frequently used for propagating zoonotic viruses. In other fields, resources like the Connectivity Map (cMAP), Human Protein Atlas (HPA), and Cancer Genomic Atlas Program (TCGA) have propelled research by providing accessible, queryable molecular datasets. A similar infrastructure for virology would enable researchers to select cell lines based on biological suitability for the virus of interest. Such databases would be particularly valuable for zoonotic pathogens, where species-specific differences can significantly impact virus behavior and experimental outcomes.

### Characterizing cell line attributes that promote viral growth

Characterizing the attributes of cell lines that influence viral growth is essential for more rational experimental design. Cell lines are often selected based on precedent or convenience rather than on traits that make them suitable for a specific virus. Developing a classification system that summarizes these traits would help researchers choose cell lines aligned with their experimental goals. Some of these attributes are known, including entry receptor expression, tolerance to cell-cycle subversion mechanisms, and absence of interferon-I response. For instance, Vero cells, which lack a type I interferon response, have historically been used in virology because this deficiency contributes to their permissiveness (Emeny and Morgan, 1979). Similarly, some retroviruses such as HIV-1 are known to arrest cell cycle in the G2/M phase (See review in Fan et al., 2018), and cell lines that can tolerate cellular stress or burden from this prolonged arrest would be ideal for growing these viruses.

In the present work, we were unable to identify datasets suitable for evaluating these cellular attributes. The expression dataset used here was derived from unstimulated, baseline cell lines, which limits interpretation—particularly because type I interferon activity is a well-characterized factor in viral infection and growth. Addressing these gaps by moving away from sporadic, incomplete datasets toward more systematic and accessible data-collection practices would enable researchers to match cell line selection with specific research aims more effectively, reduce trial-and-error, and ultimately yield more accurate biological insights.

### Leveraging biological diversity through multi-cell line approaches

As described above, optimal virus–cell line pairing depends on many factors and often requires engineering the host cell. However, when working with a poorly characterized virus with the goal of simply to amplify the virus and establish basic characteristics, starting with cell lines that are generally permissive to a broad range of viruses is a practical first ste For this reason, the field has largely converged on a few such cell lines, which has led to bias and inaccurate experimental outcomes.

One solution to this problem is identifying additional cell lines that are generally permissive for virus growth. Especially if these cell lines span different tissue types or species, researchers can select cell lines that closely match the virus’s likely biological target tissue types and reduce artifacts caused by mismatched tropism at the outset. Better yet, researchers may also choose to use multiple biologically diverse set of cell lines for those initial characterization in order to increases the probability of detecting infection and helps uncover unexpected viral phenotypes or host-specific behaviors earlier on.

To this end, we conducted an initial analysis identifying several underutilized cell lines with gene expression profiles similar to those of popular cell lines (**Figure 2B**). We selected one cell line that exhibited the most favorable combination of attributes: overall expression similarity to the popular cell lines, comparable receptor coverage, confirmed commercial availability, and documented use in the literature.

By incorporating a biologically diverse panel of permissive cell lines rather than relying on a single default cell line, researchers can more reliably capture early viral behaviors and avoid overlooking critical aspects of viral biology.

## Conclusion

Choosing cell lines to grow viruses is often the first decision made in studying viruses. While historically popular cell lines such as Vero, BHK-21 and MDCK have enabled critical discoveries, reliance on a limited set of models introduces bias and may obscure important aspects of viral biology. This concern is especially relevant for emerging and zoonotic viruses, where host-specific factors are poorly understood or unknown. To address these limitations, we propose a more systematic and diversified approach to cell line selection based on molecular characterization and biological relevance. We advocate for building molecular databases for cell lines from diverse organisms, understanding virologically relevant attributes, and encouraging the use of biologically diverse panel of cell lines. Such efforts will not only improve the reproducibility and interpretability of virology studies but also enhance our ability to identify unknown viral phenotypes, better understand viral pathogens, and ultimately prepare more effectively for emerging infectious diseases.

## Methods

### Literature review

All publications included in the literature review were retrieved from PubMed on June 23, 2023, using the search terms: *cell line AND virus AND (propagation OR amplification)*. This search yielded 6553 articles. Abstracts were screened using the PubTator tool to identify direct mentions of both a virus and a cell line, resulting in 2024 relevant publications. These abstracts were then further analyzed using ChatGPT (models GPT-3.5, GPT-4, and GPT-4o) to assess whether the primary focus of each article involved the use of cell lines for virus amplification. This process identified 1044 relevant publications. Among these, 141 publications mentioned two or more cell lines and were manually reviewed. Of those, 47 were confirmed to involve direct comparisons of virus amplification across multiple cell lines. An overview of the literature review and screening process is shown in **Figure 1A**.

### Use of external and reference datasets

For enrichment analysis, gene sets were obtained from the C5 collection of the Molecular Signatures Database (MSigDB) from the Gene Set Enrichment Analysis (GSEA) resource. From the 16228 total gene sets in this collection, 52 virus-related gene sets (by selecting gene set names containing virus or viral) containing a total of 710 genes were selected for downstream analysis.

Cell line expression data were sourced from the Human Protein Atlas (HPA; https://www.proteinatlas.org/) Specifically, the dataset labeled “RNA expression in 1206 cell lines” was downloaded, and normalized expression values (nTPM) were used for all subsequent analyses.

RNA-seq datasets for three popular cell lines (Vero E6, BHK-21, and MDCK) were retrieved from the NCBI Gene Expression Omnibus (GEO) under the following accession numbers: GSE178942, GSE93045, and GSE290599, respectively. Only samples labeled as controls were included to ensure baseline expression comparisons. Raw sequence data were downloaded from the Sequence Read Archive (SRA; https://www.ncbi.nlm.nih.gov/sra/) directly into the Galaxy analysis platform (https://usegalaxy.org/), where reads were quality-filtered, aligned to the reference genome, and count data were generated.

### Data and statistical analysis

All analyses were conducted in R (version 4.3.0). The following packages and statistical methods were used:

For the gene set enrichment analysis, the tmod package (version 0.50.13) was used. The CERNO test, which ranks gene lists and combines p-values using Fisher’s method, was applied to assess enrichment of virus-related gene sets. Enrichment scores of popular cell lines were compared against background (all kidney-derived cell lines in the HPA dataset) using a two-sided Wilcoxon rank-sum test.

The receptor coverage plot was generated based on known virus receptors as compiled by Valero-Rello et al. (2024). We have selected one cell line per tissue type that met the following criteria as good candidates for virus growth substrates:1) similar expression as the popular cell lines, 2) similar receptor coverage, 3) commercial availability, and 4) evidence of use by the research community (i.e., research publications on the cell line available).
